# Effects of Strong Earthquake on Plant Species Composition, Diversity, and Productivity of Alpine Grassland on Qinghai-Tibetan Plateau

**DOI:** 10.3389/fpls.2022.870613

**Published:** 2022-04-12

**Authors:** Hui Zuo, Hao Shen, Shikui Dong, Shengnan Wu, Fengcai He, Ran Zhang, Ziying Wang, Hang Shi, Xinghai Hao, Youquan Tan, Chunhui Ma, Shengmei Li, Yongqi Liu, Feng Zhang

**Affiliations:** School of Grassland Science, Beijing Forestry University, Beijing, China

**Keywords:** seismo-fault, alpine grasslands, vegetation composition, species diversity, plant productivity

## Abstract

Earthquakes occur frequently in fragile alpine grassland areas on the Qinghai-Tibet Plateau (QTP), but few studies have evaluated the impacts of seismo-fault of earthquake on alpine grassland vegetation diversity. In this study, we conducted a field survey of plant communities of alpine grassland along the fault zone in the 7.4 Maduo earthquake occurred on 22 May 2021. Surrounding grassland habitat far from the seismo-fault of earthquake was selected as the control. Plant community metrics around and far from seismic rupture were studied. The results showed that plant community metrics were negatively affected by seismo-fault of earthquake. Species composition around seismo-fault was being shifted from sedges-dominant into forbs-dominant. In addition, the diversity and aboveground biomass were significantly decreased around seismo-fault compared with the control. Our findings highlighted that earthquakes can cause species loss and plant community shift and finally lead to productivity reduction of alpine grassland. Additionally, forbs may be more competitive than other functional groups after the earthquake.

## Introduction

Natural disasters have profound impacts on global biodiversity (Sagar and Singh, 2005; Fattorini, 2010; Milner et al., 2013; Fraver et al., 2017; Ohbayashi et al., 2017). As a serious natural disturbance (Pickett and White, 1985; Hobbs and Huenneke, 1992), earthquake is one of the natural disasters that causes ecosystem degradation (Huang et al., 2017; Kitada et al., 2017; Tsujimoto et al., 2020; Kang et al., 2021). Such disturbance may greatly threaten the survival and competition of plant species by increasing mortality and changing habitat quality (Sousa, 1984; Lai et al., 2007; Huang et al., 2017; Ohbayashi et al., 2017; Tsujimoto et al., 2020). The magnitude and frequency of the disturbances of earthquakes can affect species diversity and its distribution (Lindenmayer et al., 2010), which leads to species loss and grassland fitness damage.

The response mechanism of plants to earthquakes is a hot topic and still remains unclear (Renaud et al., 2013; Cutter et al., 2015; Klein et al., 2019). Some studies showed that earthquakes severely damaged the structure and function of grassland ecosystems, which causes biomass decrease, diversity decline, and soil erosion (Guariguata, 1990; Allen et al., 1999; Ge et al., 2009). The study on the characteristics of plant communities after the magnitude 8.0 Wenchuan earthquake in China suggested that the earthquake can change the primary forest community into secondary grassland communities (Kang et al., 2021). In the earthquake-stricken regions, the area of grassland, forest, and wetland ecosystems were greatly reduced, with diversity and species abundance decreasing dramatically (Ouyang et al., 2008; Zhang et al., 2011). In addition, the recovery rate of grassland was the lowest among different vegetation types during the restoration process after the earthquake (Yang and Qi, 2017). Therefore, it is of great significance to understand the effects of earthquake on the structure and function of plant community in future vegetation restoration after earthquakes.

Known as “the Roof of the World” and “the Third Pole,” Qinghai-Tibet Plateau (QTP) has an area of about 2.5 million square km territory with average altitude of more than 4,000 m (Dong et al., 2020). Covering above 60% of the whole territory, alpine grassland is the dominated ecosystem and natural resource of the QTP (Dong et al., 2020). It is the most important life support system in the region and the key alpine species gene pool in the world (Dong et al., 2013). Due to the continuous continental collision between the Eurasian plate and the Indian plate, the QTP is one of the regions with strong seismic activity in the world, which is caused by significant tectonic loads and crustal deformation (Chen et al., 2021). According to the previous study, the frequency of earthquakes is higher in the border regions between the lithospheric plates (Fattorini et al., 2018). Alpine region is a quake-prone area, and it is sensitive to external environmental change (Dong et al., 2020). Fractures are formed after earthquake, which can obviously cause negative effects on grassland. To date, we still know less about how alpine grassland ecosystems, especially grassland plant community, which are fairly sensitive to external disturbance, respond to the earthquake.

Generally, grassland ecosystems can resist external disturbance through natural adjustment, yet vegetation restoration methods can help to accelerate the restoration process (Alonso et al., 2006). The maintenance of grassland community diversity and grassland structure mainly depends on the renewal and restoration of plants (Chesson, 2000). Assuming the plants are not severely damaged, they are likely to be vegetatively propagated and begin to recover from the remaining buds (Klimešová and Klimeš, 2007; Liew et al., 2013), especially in grasslands where perennial grasses are the main building species (Benson and Hartnett, 2006). In addition, natural grassland soil contains a large number of germinating plant seeds, which play an important role in the restoration and succession of plant communities (King, 2007; Liu et al., 2009; Latzel et al., 2011). Therefore, we investigated the status of vegetation composition, plant diversity, and biomass of alpine grassland in the earthquake sites in Maduo County of China 4 months after magnitude 7.4 earthquake occurred on 22 May 2021 in this study. We hypothesized the following: (1) the earthquake can redistribute species abundances of alpine grassland along the seismo-faults; (2) the earthquake can decrease the plant diversity and productivity of the alpine grassland by damaging morphology of the local habitats. The purpose of this study is to clarify how will alpine grassland plant community (species composition, diversity, and aboveground biomass, etc.) responded to earthquake in a short period. It is expected to provide insights for quickly detecting the effects of earthquakes on alpine grassland ecosystem and to rationally promote recovery of alpine grassland ecosystems disturbed by the earthquake on the QTP.

## Materials and Methods

### Study Site

The study sites were located in Maduo county (96°50′-99°20′E, 33°50′-35°40′N), northwest of Guoluo Tibetan Autonomous Prefecture, Qinghai Province, China. Maduo County has a unique geographical location and climatic conditions, which is an overlap area between the alpine ecological fragile area and the national key ecological function area. The annual average temperature is 4.1°C, and the annual average precipitation is 303.9 mm with large annual variation. The vegetation is mainly alpine grassland (alpine steppe and meadow), and the soil is sandy loam. There are mainly 30 families, 140 genera, and 429 species of forages, such as Cyperaceae, Compositae, and Gramineae.

Maduo county is situated at the margin fault of the Bayanhar block, where earthquakes occur frequently (Wang et al., 2021). On 22 May 2021 earthquake occurred on a secondary fault within the active Bayanhar block, which creates several seismic fault zones. It was the largest earthquake on the QTP in the past two decades, which causes 6.3 billion yuan in damage (Chen et al., 2021). According to the China Earthquake Information Network (http://www.csi.ac.cn/), Maduo earthquake has the focal depth of 17 km, and the average altitude of 4,200 m within 10 km of the epicenter. Up to date, many scholars have kept on studying on the occurrence mechanism and induced geohazards of the 2021 Maduo earthquake (Chen et al., 2021; Gao et al., 2021; Wang et al., 2021; Zhu et al., 2021). However, we still know less about how will plant community responds to the earthquake in alpine grassland.

### Field Surveys

**Figure 1:**
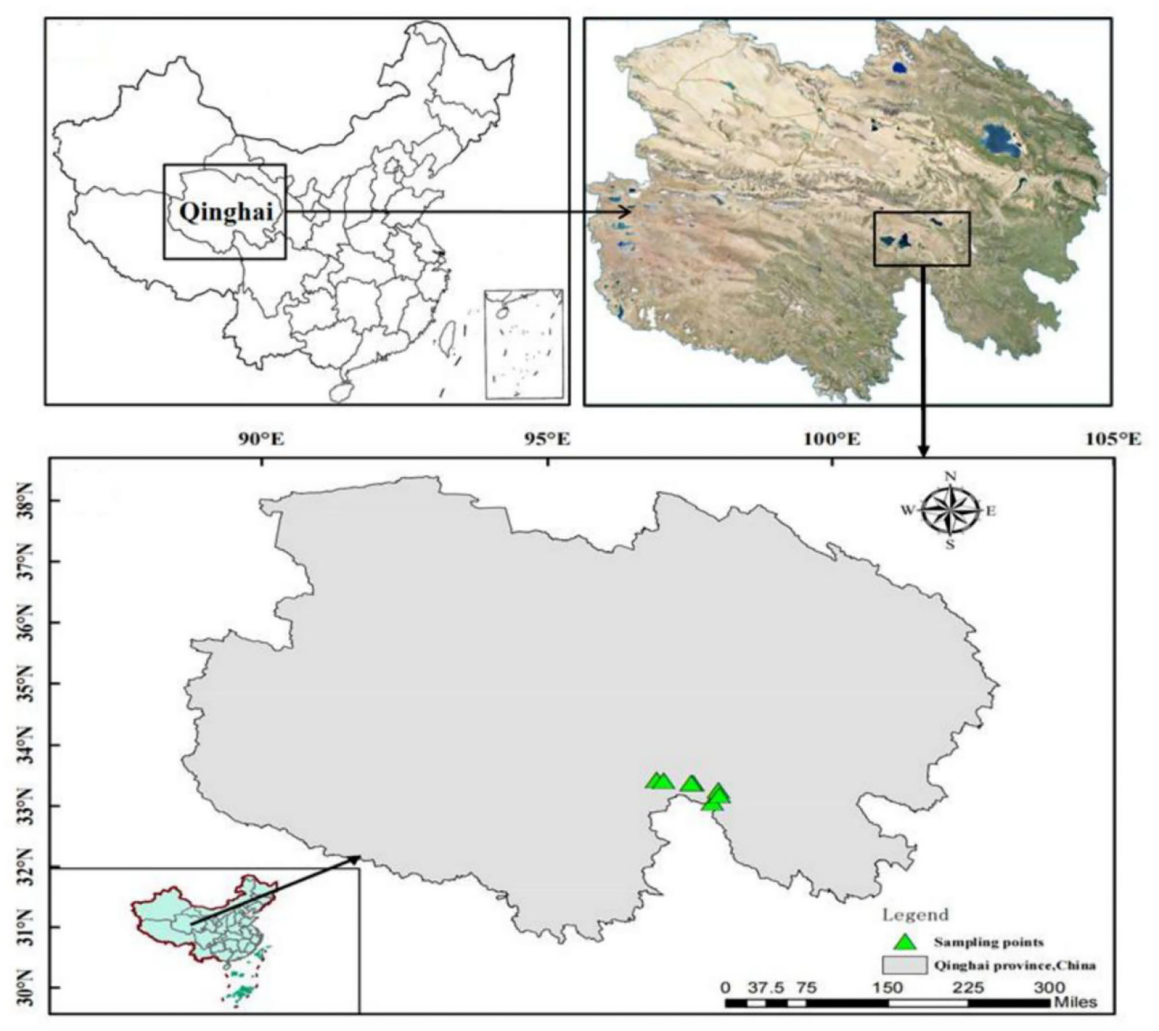
Location map of sampling sites.

Field investigation was conducted in the early September 2021. After investigation, according to the surface fault distribution of the 22 May Maduo earthquake, we selected seven typical seismo-faults as the sampling sites (Figure 1, Table 1). The paired sites far from the seismo-fault were sampled as the controls to identify changes in plant composition, diversity, and productivity of alpine grassland with the strong earthquake and to explore the response of vegetation in the short-term post-earthquake areas of the QTP. The average elevation of the sampling sites was about 4,200 m. We chose the sites about 3 m close to the seismo-fault as the impact sites of the earthquake fault. Additionally, the sites about 30 m away from the seismo-fault with the same slope and aspect of the impact sites were used as the control sites. In the seismic fault site and the control site of each sampling pair, we randomly placed three 1 × 1 m quadrats, respectively, to record the height, abundance, and coverage of both individual species and plant community. In addition, we recorded the latitude and longitude of each sampling pair with the Global Positioning System (GPS). After the survey, the aboveground parts of plants were collected and taken back to the laboratory for oven drying at 70°C to a constant weight.

**Table 1:** The location of the sample points.

	**I**	**II**	**III**	**IV**	**V**	**VI**	**VII**
CK	35° 3'45”N, 98°44'14” E	34° 44'21” N, 97° 45'23” E	34° 43'27”N, 97° 55'8” E	34°12'19” N, 98°40'38” E	34° 32'55”N, 98° 48'56”E	34°24'40” N, 98°50'0”E	34°41'53”N, 98° 0'5”E
Seismo-fault	35°3'57”N, 98°44'15”E	34°44'19”N, 97° 45'22”E	34° 43'25”N, 97°55'6”E	34° 42'22”N, 98°40'26”E	34°35'25”N, 98° 48'12”E	34°34'40”N, 98° 50'3”E	34°41'50”N, 98°0'7”E

*CK, Areas about 30 meters away from the seismic fault zones; Seismo-fault, Areas close to seismic fault zones*.

### Data Analysis

We divided the sampled plants into five major functional groups: grasses, sedges, legumes, forbs, and others. The important values (IVs) of plant species and functional groups were calculated according to the following formula:


(1) $$\begin{array}{r} \text{IV}=\left(\text{Rh}+\text{Rc}+\text{Ra}\right)/\;3 \end{array}$$

where Rh, Rc, and Ra refer to the relative height, relative coverage, and relative abundance.

We classified the alpine plant communities using the R package and calculated Shannon–Wiener index, Simpson index, inverse Simpson index, species richness index (S), and species evenness index (J).

We used SPSS 26.0 to analyze the data and performed a two-tailed *t*-test and two-way ANOVA on the plant community composition, diversity indices, and above-ground biomass (AGB) of the control and seismic fault zones. The R package (4.1.2) was used for non-metric multidimensional scaling (NMDS) of species composition between the seismic fracture sites and the control sites, and GraphPad Prism 9.3 was used for drawing.

## Results

### Effects of Seismic Fault on Species Composition of Alpine Grassland Plant Community

**Figure 2:**
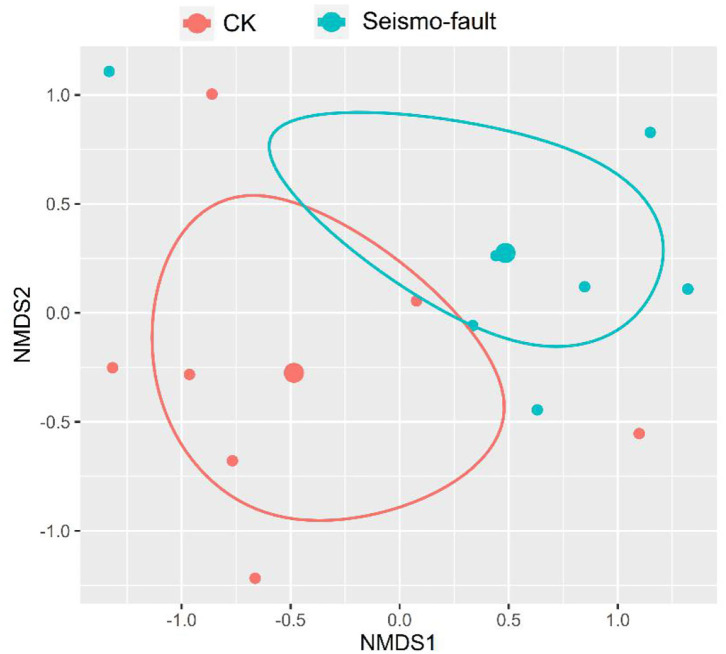
Non-metric multidimensional scaling plot of the species composition between the seismic fracture sites and the control sites. Dots of the same color come from the same group. Each small dot represents a site replication, and each large dot represents the mean value of sites replications. Circles indicate the 95% confidence of the mean value.

We found that there were 45 species belonging to 37 genera and 20 families on the Maduo earthquake zone (Supplementary Table 1). Among them, 4 plant species belonged to Gramineae, 6 plant species belonged to Cyperaceae, 1 plant species belonged to Leguminosae, and other plants were 10 species of Compositae and 3 species of Rosaceae. Far from the seismo-faults, we also found two small shrubs, *Oplopanax elatus* of the Araliaceae and *Salix cupularis* of the Salicaceae. The NMDS analysis showed that the species composition in the seismic fault sites and the control sites were obviously different (Figure 2). In the control site, 38 plant species belonging to 30 genera and 18 families were recorded, and the common families were Gramineae, Cyperaceae, and Compositae. In the seismo-fault sites, 27 species of plants belonging to 25 genera and 14 families were recorded, and the common families were Gramineae, Compositae, and Polygonaceae. These results indicated that the seismic rupture caused a significant reduction in the number of species, with about 22, 16, and 29% decline at family, genera, and species levels, respectively. Sedge species significantly declined among all five functional groups in seismo-fault sites compared with the control.

### Effects of Seismic Faults on Structure of Alpine Grassland Plant Community

At the species level, we found that the IVs of different component species in the plant community changed significantly between the seismic fracture sites and the control sites (we listed specific changes in species IVs at seven sampling sites in Supplementary Figures 2A–8A), due to the influence of seismic fracture. Combining the unified analysis of the seven sampling sites, we can clearly see that *Kobresia humilis* and *Kobresia pygmaea* were affected by the seismo-fault, and their IVs dropped greatly (Table 2 and Supplementary Figure 1). Except for *Leymus secalinus*, the IVs of other plant species in Gramineae all decreased significantly. The legume plant *Oxytropis ochrocephala* and shrub plants *Salix cupularis*, as well as *Oplopanax elatus*, disappeared, and the IVs of forb plants *Polygonum sibiricum* and *Glaux maritima* increased remarkably. Among all species, the IV of *Kobresia humilis* decreased the most, whereas the IVs of *Polygonum sibiricum* and *Glaux maritima* increased the most. Earthquake rupture made the dominant species of the community shift from *Kobresia humilis* (IV 0.156) and *Kobresia pygmaea* (IV 0.102) to *Polygonum sibiricum* (IV 0.223), *Glaux maritima* (IV 0.165), and *Artemisia capillaris* (IV 0.109).

**Table 2:** Species importance values under the influence of earthquake rupture.

	**CK**		**Seismo-fault**	
*Kobresia humilis*	+	0.156252233		
*Kobresia pygmaea*	+	0.102136647	+	0.055289472
*Carex scabrirostris*	+	0.088250531	+	0.081428420
*Artemisia capillaris*	+	0.061800631	+	0.108706828
*Poa pratensis*	+	0.045944745	+	0.019881392
*Salix cupularis*	+	0.035319345		
*Glaux maritima*	+	0.034876339	+	0.164660069
*Leymus secalinus*	+	0.033391641	+	0.075255887
*Leontopodium leontopodioides*	+	0.032933751	+	0.003692253
*Agropyron cristatum*	+	0.030545360	+	0.007587376
*Polygonum sibiricum*	+	0.030284918	+	0.222581596
*Heteropappus altaicus*	+	0.029762618		
*Gentiana straminea*	+	0.028815976	+	0.034499363
*Potentilla multifidi*	+	0.027936715		
*Kobresia tibetica*	+	0.026920004		
*Scirpus triqueter*	+	0.024095103		
*Lancea tibetica*	+	0.019389092	+	0.046746379
*Lxeris chinensis*	+	0.017023095	+	0.009620035
*Arenaria pulvinata*	+	0.016414170	+	0.010183098
*Allium przewalskianum*	+	0.016232041	+	0.023687581
*Oxytropis ochrocephala*	+	0.013720787		
*Plantago asiatica*	+	0.01357746	+	0.004683053
*Scirpus distigmaticus*	+	0.013163589		
*Triglochin palustre*	+	0.013110319		
*Rhodiola rosea*	+	0.012717770	+	0.007874367
*Potentilla bifurca*	+	0.011526361	+	0.009075500
*Lagotis brachystachya*	+	0.009465062		
*Oplopanax elatus*	+	0.008236880		
*Puccinellia distans*	+	0.007798093	+	0.003962308
*Adenophora capillaris*	+	0.007131411		
*Allium sikkimense*	+	0.006359003		
*Triglochin maritimum*	+	0.005099940		
*Cirsium japonicum*	+	0.005048336	+	0.005219238
*Artemisia frigida*	+	0.004553808	+	0.043194969
*Potentilla acaulis*	+	0.004302895		
*Taraxacum mongolicum*	+	0.002666353		
*Dracocephalum heterophyllum*	+	0.001623062		
*Saussurea stella*	+	0.001573915		
*Aster tataricus*			+	0.005048372
*Limonium aureum*			+	0.001948876
*Ranunculus membranaceus*			+	0.002662533
*Rheum nanum*			+	0.007941323
*Saussurea japonica*			+	0.017003277
*Thalictrum petaloideum*			+	0.014379470
*Comastoma pulmonarium*			+	0.013186966

*The “+” indicates that the species existed in this area, and the important value is written after it. The vacancy indicates that the species absent in this area. The highest species important values in the seismic fracture sites and the control sites areas are marked in bold red*.

**Figure 3:**
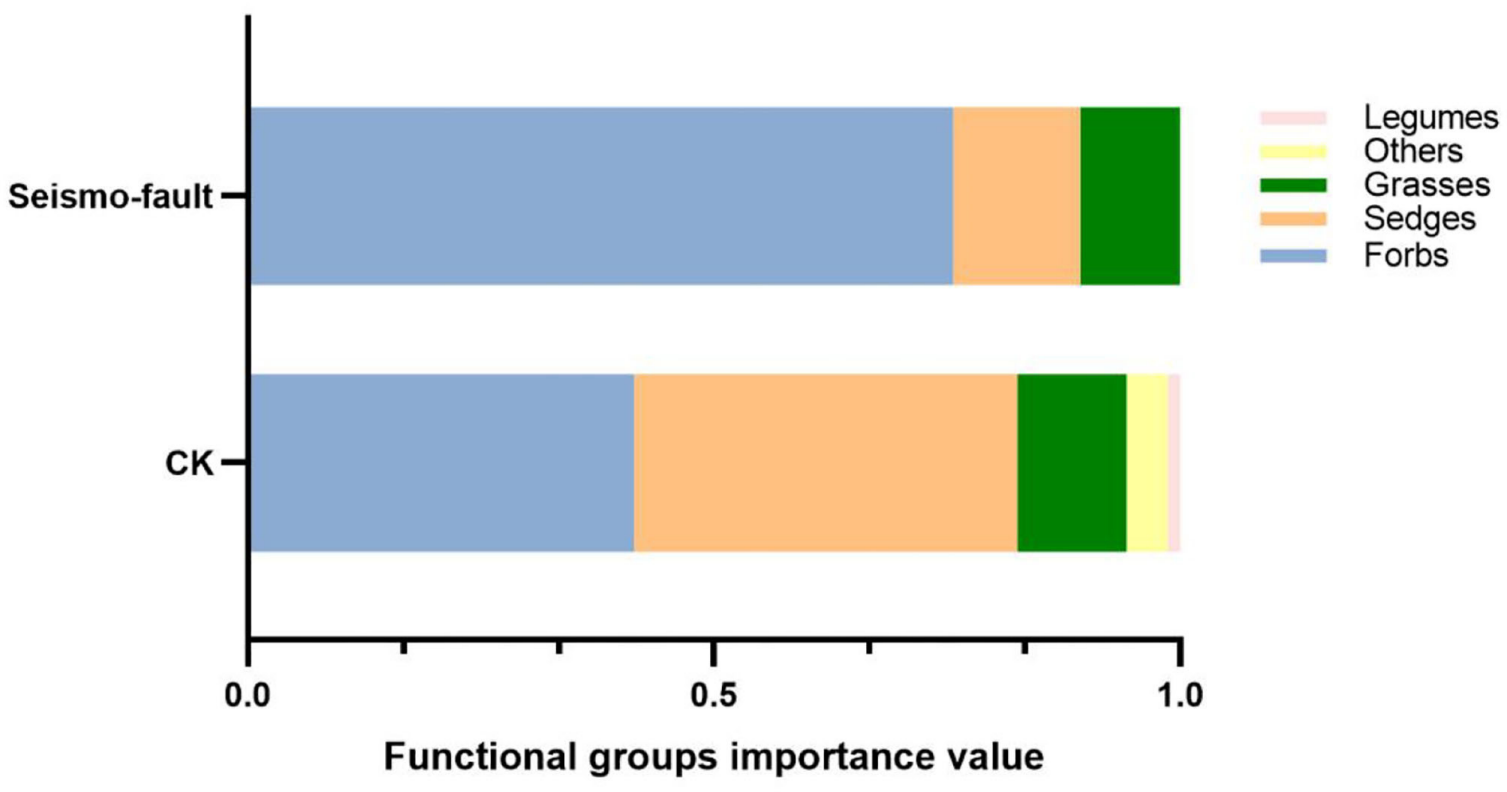
The importance values of functional groups in different seismic regions.

At the functional group level, the IVs of different functional groups of plants varied greatly due to seismo-fault (Supplementary Figures 2B–8B). In addition to the sixth sampling site where all the functional groups were forbs, the IVs of sedges and legumes decreased in the other sampling sites and the proportion of forbs increased significantly in comparison with the control sites. Meanwhile, except for the second and fifth sampling sites where the IVs of the functional groups of grasses increased slightly, the IVs of the other sampling sites containing grasses decreased significantly. Overall, seismo-fault significantly affected the composition of sedges, legumes, and forbs functional groups (Supplementary Table 2), which reduces the IVs of sedges, grasses, and legumes by 0.274, 0.011, and 0.014 (Figure 3). As for the community structure in seismo-fault sites, the dominated functional groups changed from co-dominated groups of forbs (IV 0.422) and sedges (IV 0.411) to solely-dominated group of forbs (IV 0.757).

### Effects of Seismic Fracture on Species Diversity of Alpine Grassland Plant Community

**Figure 4:**
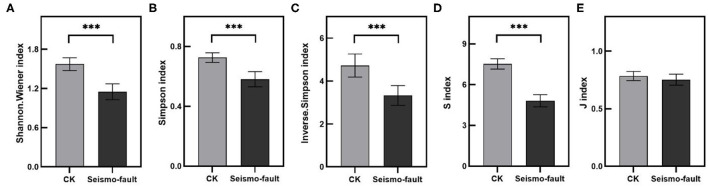
Diversity indices in different seismic regions. **(A)** Shannon–Wiener index, **(B)** Simpson index, **(C)** inverse Simpson index, **(D)** species richness index, and **(E)** species evenness index. Vertical bars represent the SE of mean. Asterisks on the SE bars show significant differences between the control (gray bars) and seismo-fault (black bars) (***p < 0.001).

As it can be seen from Supplementary Figures 2C–8C, there were significant differences in the diversity indices for the plant community between the seismo-fault sites and the control. At the same time, different sampling sites and the joint action of sampling sites and the seismo-fault also had a significant impact on the diversity of plant communities (Supplementary Table 3). In most communities, Shannon–Wiener index, Simpson index, inverse Simpson index, and species richness index in the control sites were higher than those in the seismic fault sites. In samplings sites II and VI, the differences in plant diversity indices were very distinct (Supplementary Figures 3C, 7C). The species richness of sampling sites IV and V was also markedly higher than that of the control sites (Supplementary Figures 5C, 6C). However, there was no clear change in the species evenness index for most sampling sites, except for sampling site VI, where the species evenness of seismo-fault site was observably lower than that of the control site (Supplementary Figure 7C). In general, the Shannon–Wiener index, Simpson index, inverse Simpson index, and species richness index were significantly decreased by seismo fracture, while the species evenness index was nearly not affected by the seismo-fault (Figure 4), but at different sampling sites, the differences were obviously different (Supplementary Table 3, Supplementary Figures 2C–8C).

### Effects of Seismic Fracture on Aboveground Biomass of Alpine Grassland Plant Community

**Figure 5:**
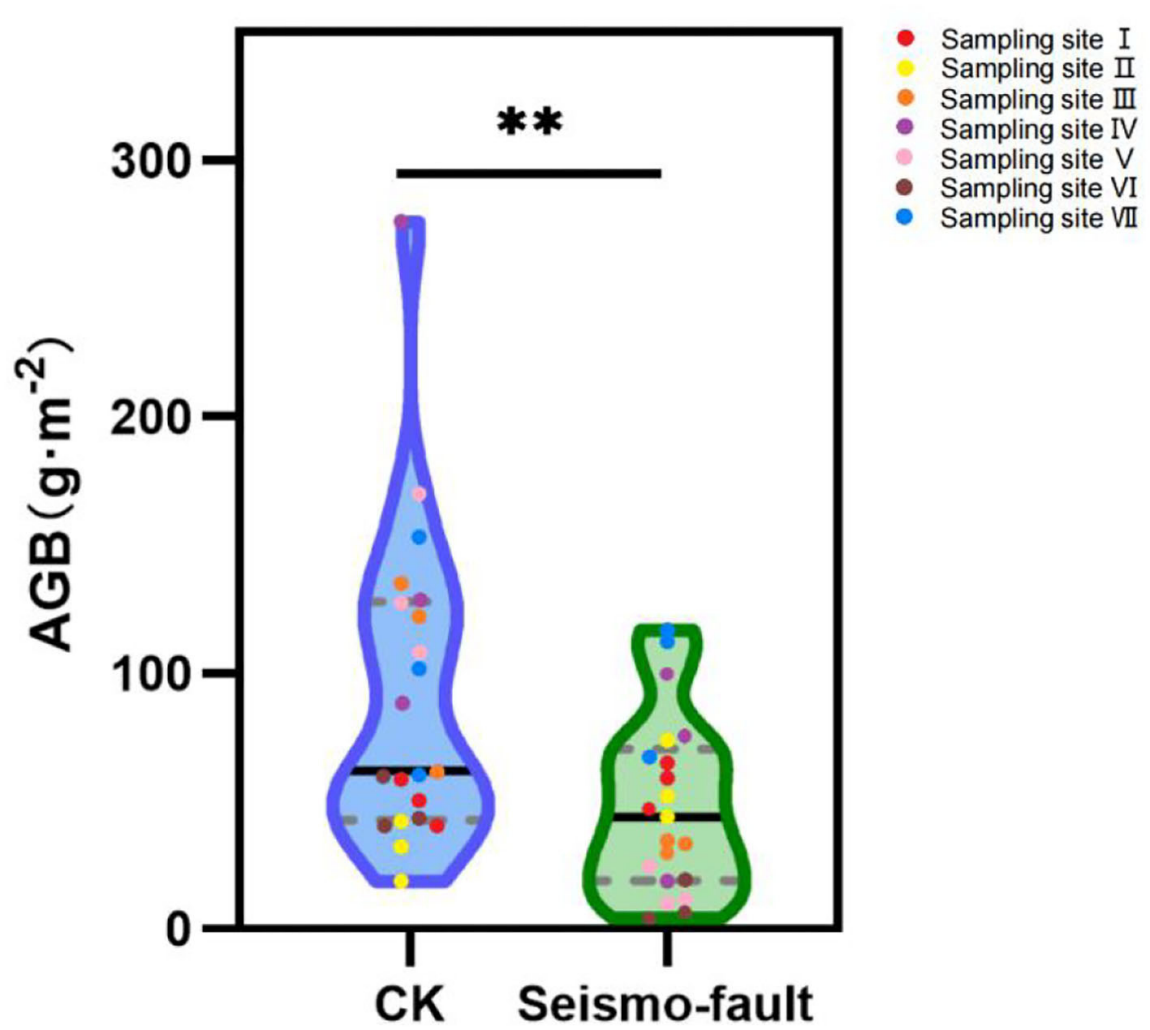
Aboveground biomass in different seismic regions. Asterisks indicate differences among treatments (**p < 0.01). Different colors represent different sampling sites.

The earthquake fault obviously impacted the aboveground biomass of alpine grassland plant community. It is evident from Figure 5 that although the aboveground plant biomass of different sampling sites was different, the aboveground plant biomass near the seismic fault was significantly lower (*p* < 0.01) than that of the control, which indicated that the grassland productivity was clearly reduced by the earthquake.

## Discussion

### Earthquake Changed Species and Functional Groups Composition

As a typical catastrophic disturbance event, earthquake has a profound impact on vegetation and terrestrial ecosystem (Yin et al., 2009; He et al., 2011; Wang et al., 2014). For example, the Wenchuan M*w* 8.0 earthquake in 2008 damaged the species diversity and richness of trees and shrubs (Zhang et al., 2011). More than 80% of the bamboo in a landslide area died after a M*w* 7.3 earthquake hit Songpan County in China in 1976 (Kleiman and Seidensticker, 1985). Our results also showed that seismo-fault obviously reduced species richness, changed species composition, and made community shift from sedges-dominant to forbs-dominant. The responses of different functional groups to seismic fracture were not consistent. The importance values of sedges and legumes decreased, and the proportion of forbs increased apparently, which suggests that the seismo-fault may result in a degenerating process, and such degradation may rapidly erode the ecosystem services that alpine grasslands provide (Harris, 2010; Dong et al., 2020). It is generally believed that the healthy alpine grassland is usually dominated by grasses and sedges (Wang et al., 2014), while original plant community is gradually replaced by the forb community with the increase of grassland degradation (Wang et al., 2014). For the alpine grassland of QTP, seismo-fault caused the disappearance of *Kobresia humilis* and greatly increased the importance values of *Polygonum sibiricum* and *Glaux maritima*, which indicates that sedge species might be much more sensitive to earthquake effects than other functional groups. This phenomenon can be attributed to species-specific biological characteristics and adaptation mechanism. Changing the reproductive strategies of plants is an appropriate strategy to cope with environmental changes (Hedhly et al., 2009). Forbs usually have rhizomes or tubers, as well as developed root systems, which might help them to better adapt to the stress environment. Additionally, after earthquake, soil structure (Matsuda et al., 2016), nutrients (Guo et al., 2013), moisture (Oommen et al., 2013), and microbial composition (Chao et al., 2021) are negatively altered. Forbs have a higher nutrient use efficiency than sedges and grasses, which makes it easier to become the dominant species as the earthquake can cause soil erosion (Zhang et al., 2020).

### Earthquake Decreased Plant Community Diversity and Productivity

Plant diversity is an ecological index that can best reflect the degree of ecosystem fitness (Loreau et al., 2001). Aboveground plant biomass is a comprehensive index reflecting plant growth status and adaptability (Xu et al., 2015; Sanaei et al., 2018). The response of plant biomass and diversity to environmental changes can reflect the resilience of plant communities (Zhang et al., 2019; Dong et al., 2020). Through these indexes, we can have a better understanding of the post-earthquake vegetation restoration situation before adopting different restoration methods. According to Grime (2001), herbages are the pioneer plants of the ecosystem, with strong adaptability, wide ecological niche, and timely adjustment in response to habitat changes. In this study, the Shannon–Wiener index, Simpson index, inverse Simpson index, and species richness index were significantly reduced by seismo-fault, and the level of biodiversity was significantly decreased. From the perspective of functional types, species-rich communities exhibit different functional traits and resource acquisition strategies among species, which often leads to higher functional diversity which is a good predictor of resistance (Byun et al., 2013). Due to the unique characteristics of grasslands, there is a strong relationship between species diversity and plant productivity (Kwaku et al., 2021). Diversity stabilizes community and ecosystem processes, that is, the higher the diversity, the more stable the community biomass (Bai et al., 2001). This is similar to our conclusion that the level of diversity near the seismic fracture zone was reduced, and the biomass also showed a very significant decline. Maintaining diversity and productivity is a central goal in managing ecosystems worldwide (Bai et al., 2001; Grace et al., 2016). In addition, the previous study has pointed out that plant diversity and productivity always have a close relation with soil nutrient status (Qiu et al., 2018). Therefore, soil nutrient imbalance induced by earthquake might be the main cause of grassland diversity and productivity decrease (Hooper et al., 2005; Reynolds and Haubensak, 2009; Qiu et al., 2018).

## Conclusion

In this study, we found that earthquake can negatively affected alpine grassland community, which causes diversity loss and productivity reduction, as well as shifting plant community to forbs-dominant. Though experimental period was short, results were robust between control and the area near seismic fault. The responses of different species and functional groups were different. Sedge species were much more sensitive to earthquake than the grasses and forbs species. The whole plant community tended to shift from the sedges and forbs co-dominated to forbs-dominated near the seismic fault. At the same time, plant species diversity and productivity of alpine grassland plant community were obviously decreased near the seismic fault. Profound negative effects on plant community were obvious near the seismic fault, posing a big threat to grassland ecosystem health. Therefore, more attention should be paid to the effects of earthquake on fragile alpine grassland ecosystem in the future. Additionally, plant species composition, diversity, and productivity can be selected as the important indicators to judge the quick ecological impacts of earthquake on the alpine grassland. Nevertheless, a longer period and multiscale field investigation are urgently needed to testify such short-term responses in the future. In addition, relations between plant community shift and soil properties change after earthquake should also be considered in the future to better understand the reasons for the shift of plant community structure after earthquake.

## Data Availability Statement

The raw data supporting the conclusions of this article will be made available by the authors, without undue reservation.

## Author Contributions

SD, HShe, and HZ planned and designed this study. HShe, SW, FH, RZ, ZW, HShi, XH, YT, CM, SL, YL, and FZ helped to conduct the experiments. SD and HShe revised this manuscript. HZ analyzed the data and wrote this paper. All authors contributed to the article and approved the submitted version.

## Funding

This study was funded by the Second Tibetan Plateau Scientific Expedition and Research Program (2019QZKK0307), National Key R&D Program of China (2021FED1124000), and National Natural Science Foundation of China (U20A2007-01, 72050001).

## Conflict of Interest

The authors declare that the research was conducted in the absence of any commercial or financial relationships that could be construed as a potential conflict of interest.

## Publisher's Note

All claims expressed in this article are solely those of the authors and do not necessarily represent those of their affiliated organizations, or those of the publisher, the editors and the reviewers. Any product that may be evaluated in this article, or claim that may be made by its manufacturer, is not guaranteed or endorsed by the publisher.
