# Supplementary material for: Effects of Strong Earthquake on Plant Species Composition, Diversity, and Productivity of Alpine Grassland on Qinghai-Tibetan Plateau
Source: Front Plant Sci. 2022 Apr 12;13:870613. doi: 10.3389/fpls.2022.870613 (PMC9039666; doi:10.3389/fpls.2022.870613)
Supplement: Supplementary file 1 [file Data_Sheet_1.docx]

Supplementary Material

# Supplementary Figures and Tables

## Supplementary Figures


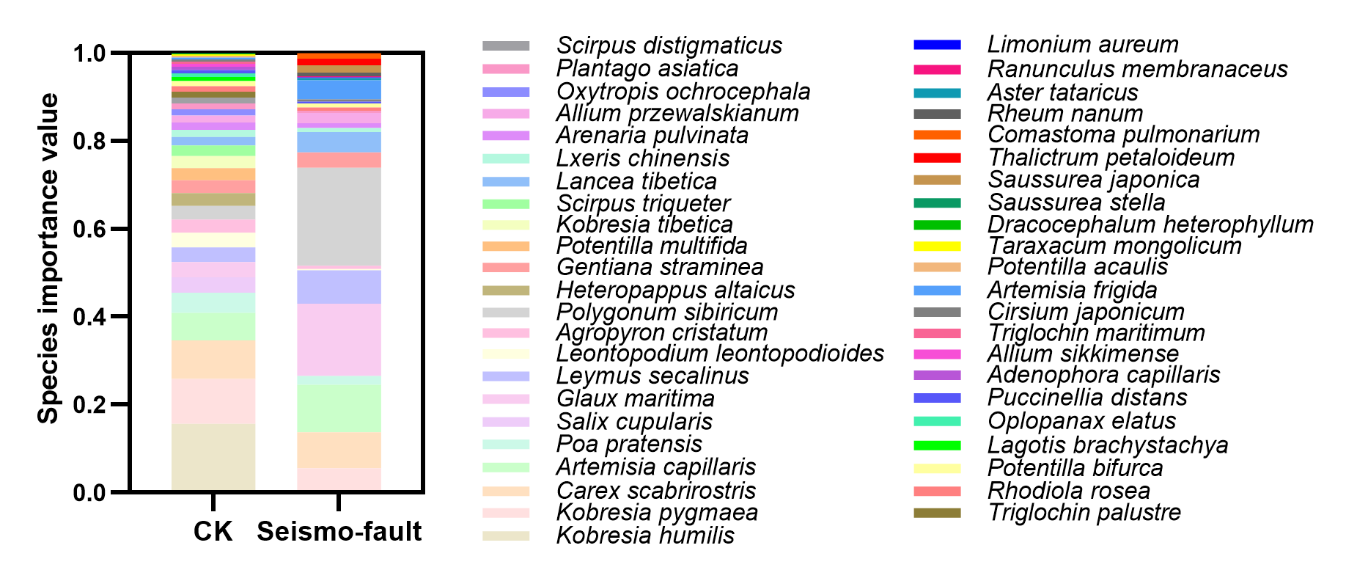


**FIGURE S1|** Species importance values in different seismic regions.


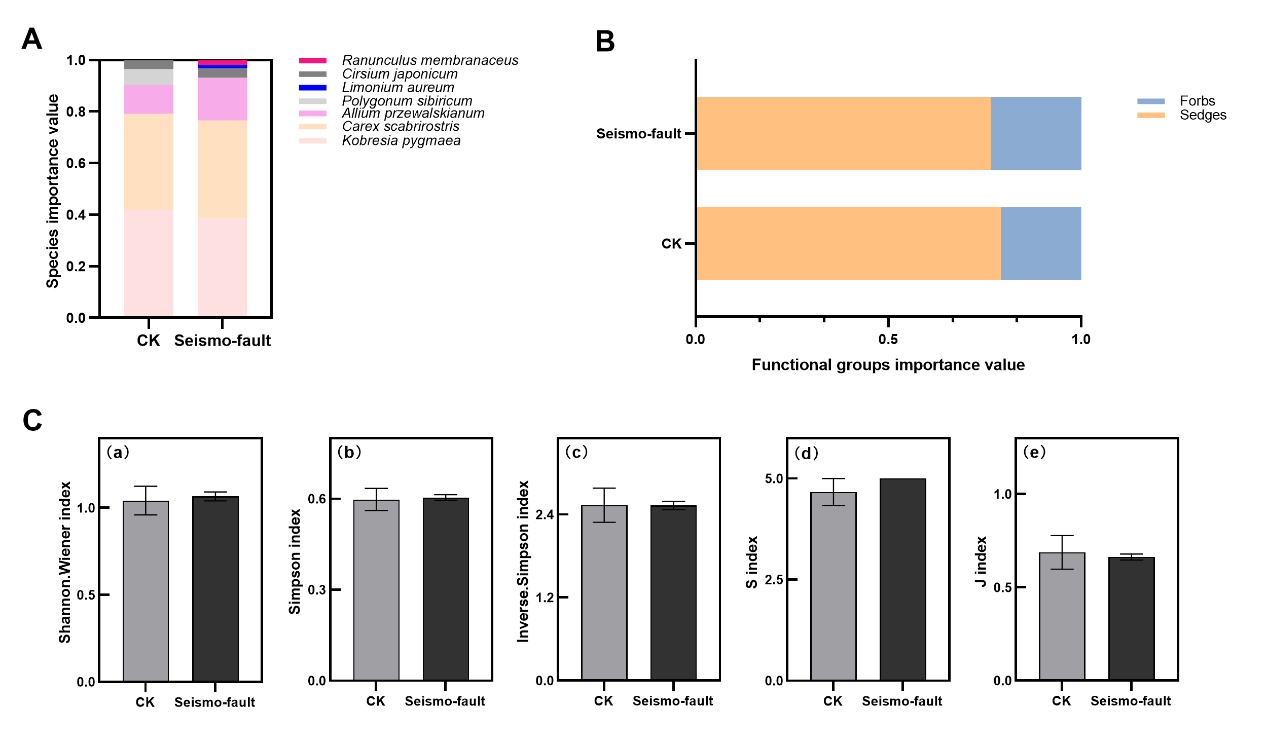


**FIGURE S2|** Effects of seismic faults on species and communities at sampling points Ⅰ. The **(A)** species importance values, **(B)** functional groups importance values, **(C)** diversity indices in the sampling points Ⅰ. Vertical bars represent the standard error (SE) of mean (n=3).


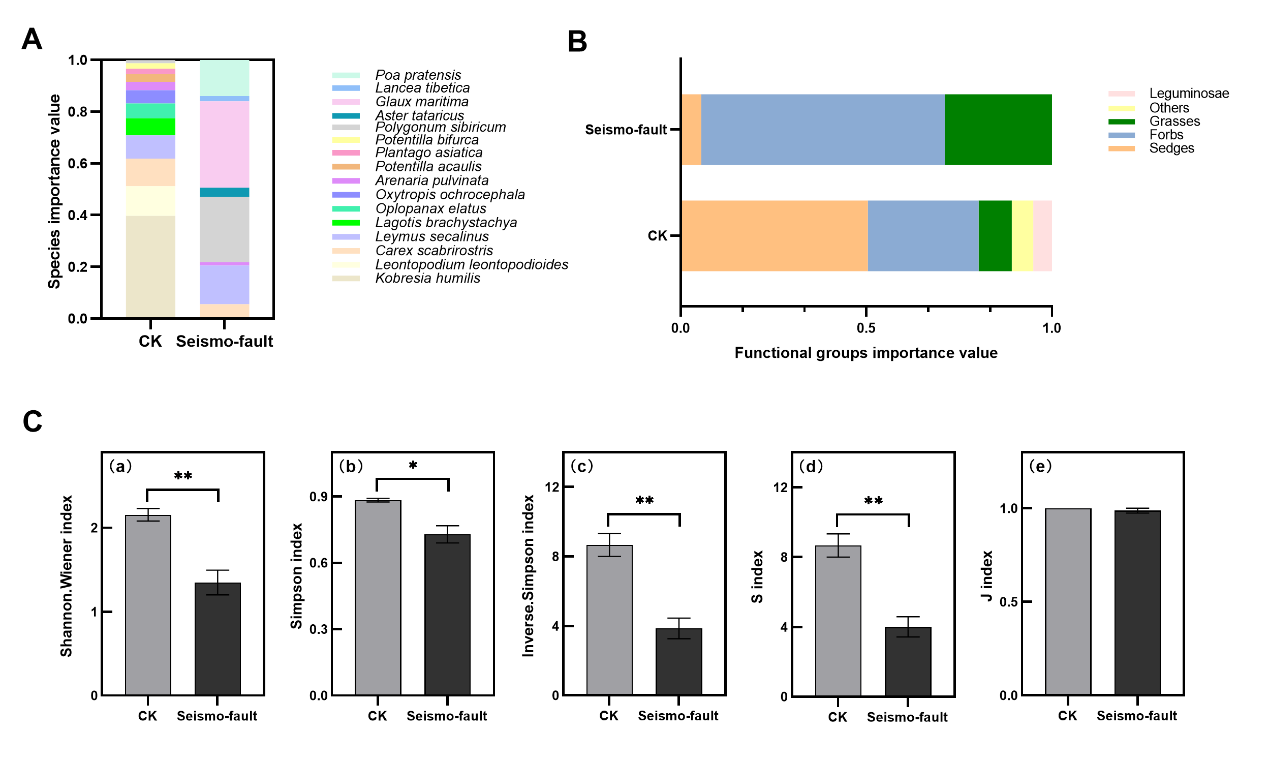


**FIGURE S3|** Effects of seismic faults on species and communities at sampling points Ⅱ. The **(A)** species importance values, **(B)** functional groups importance values, **(C)** diversity indices in the sampling points Ⅱ. Vertical bars represent the standard error (SE) of mean (n=3). Asterisks on the SE bars show significant differences between the control (grey bars) and seismo-fault (black bars) (**P*<0.05, ***P*<0.01).


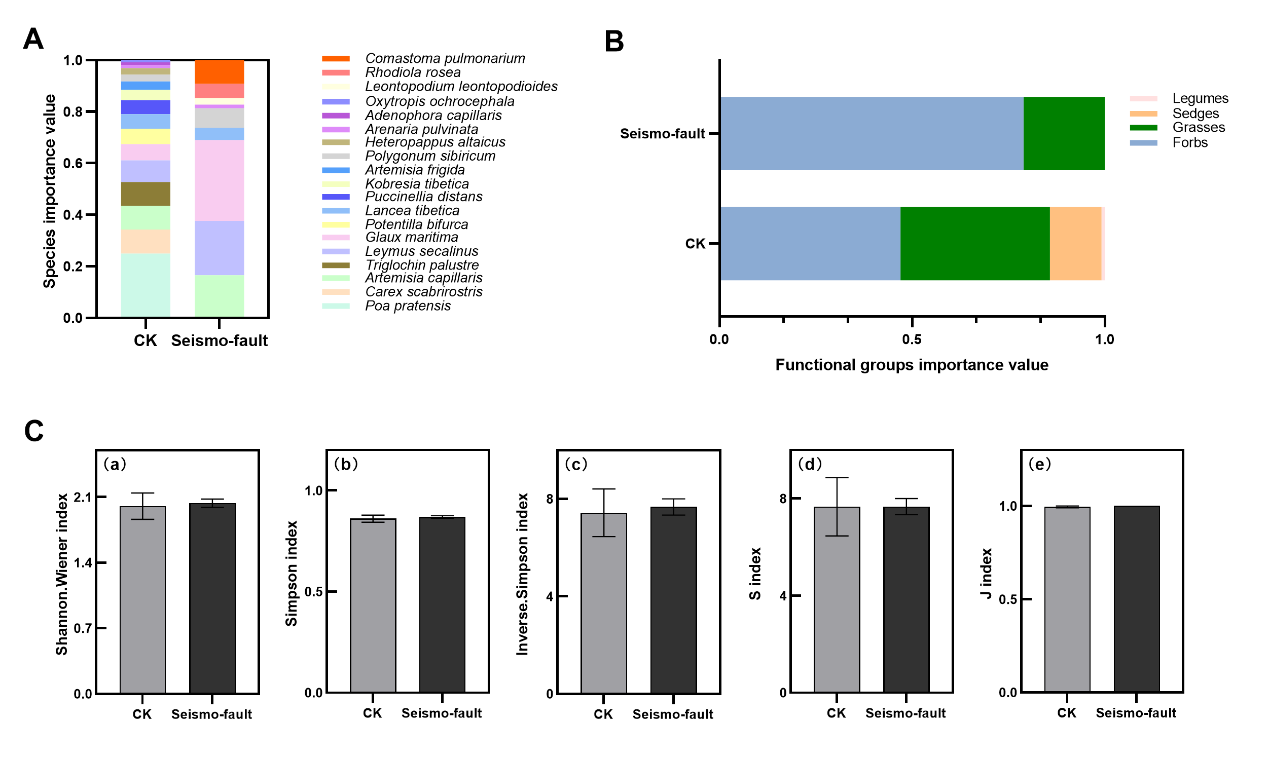


**FIGURE S4|** Effects of seismic faults on species and communities at sampling points Ⅲ. The **(A)** species importance values, **(B)** functional groups importance values, **(C)** diversity indices in the sampling points Ⅲ. Vertical bars represent the standard error (SE) of mean (n=3).


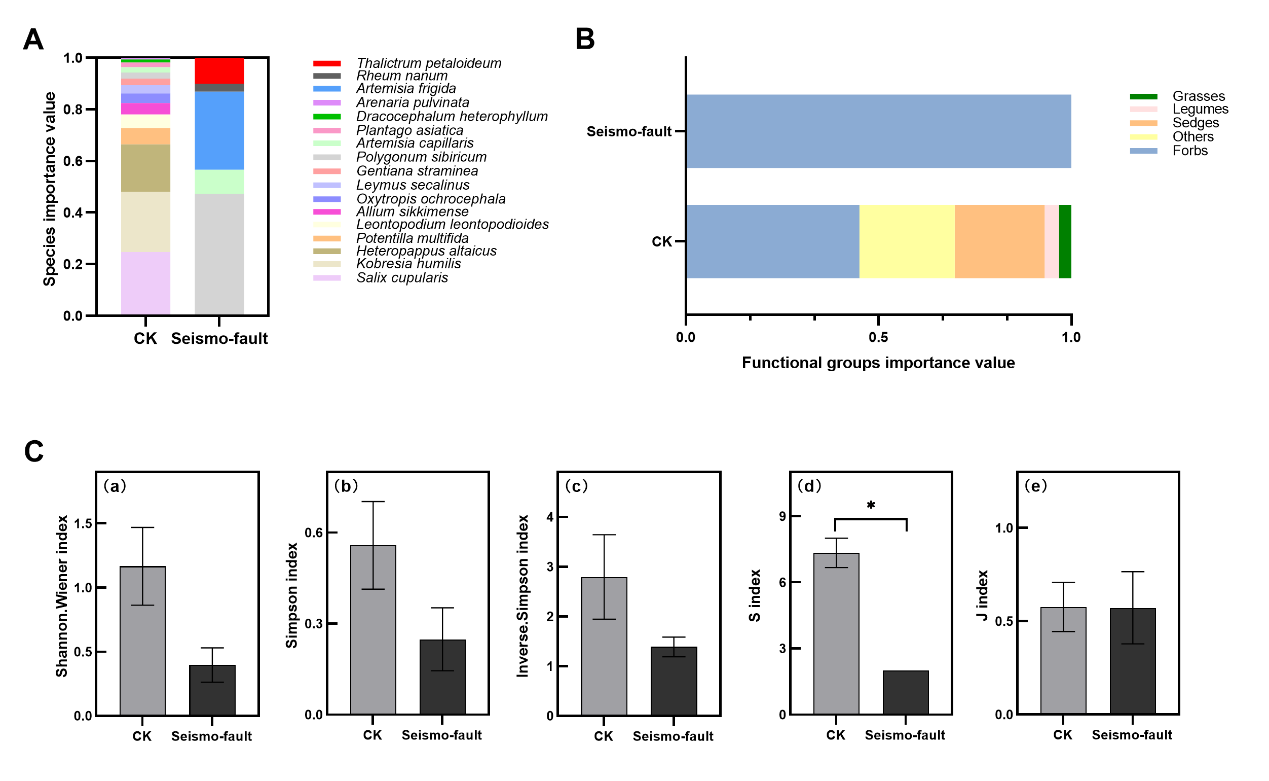


**FIGURE S5|** Effects of seismic faults on species and communities at sampling points Ⅳ. The **(A)** species importance values, **(B)** functional groups importance values, **(C)** diversity indices in the sampling points Ⅳ. Vertical bars represent the standard error (SE) of mean (n=3). Asterisks on the SE bars show significant differences between the control (grey bars) and seismo-fault (black bars) at **P*<0.05.


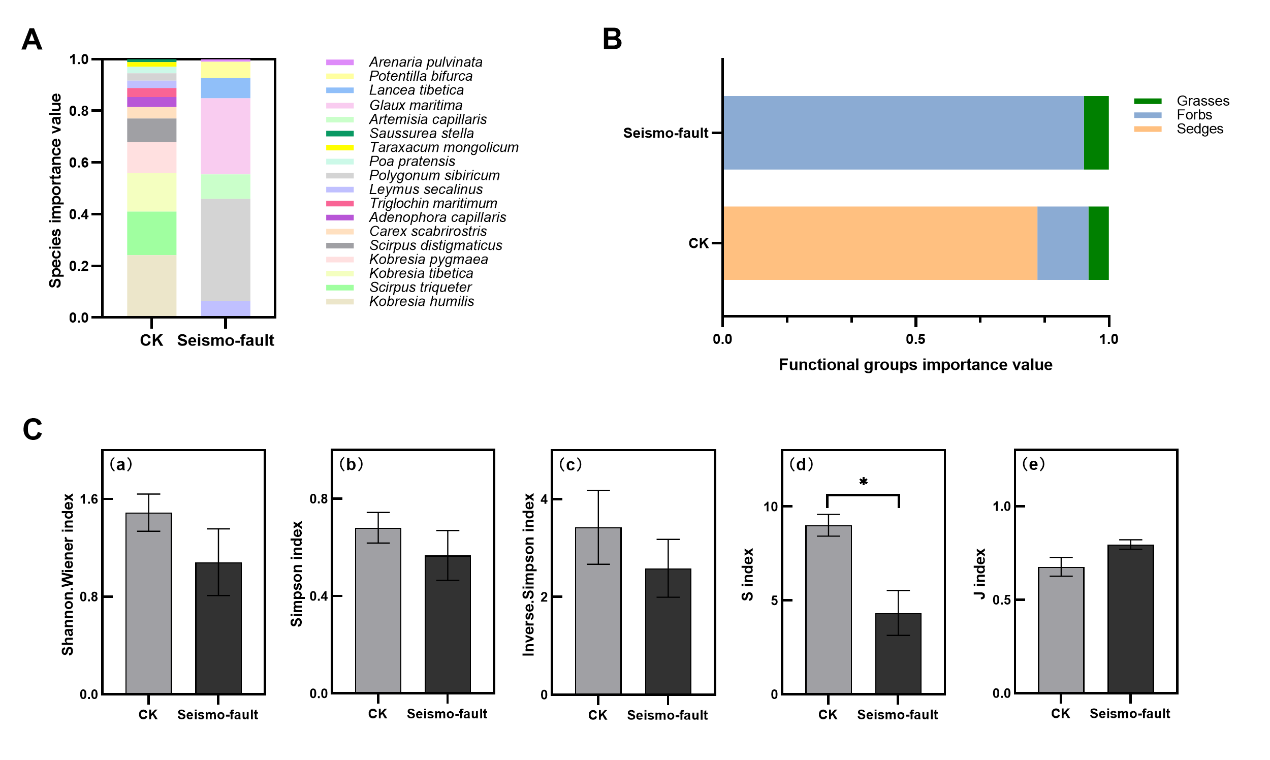


**FIGURE S6|** Effects of seismic faults on species and communities at sampling points Ⅴ. The **(A)** species importance values, **(B)** functional groups importance values, **(C)** diversity indices in the sampling points Ⅴ. Vertical bars represent the standard error (SE) of mean (n=3). Asterisks on the SE bars show significant differences between the control (grey bars) and seismo-fault (black bars) at **P*<0.05.


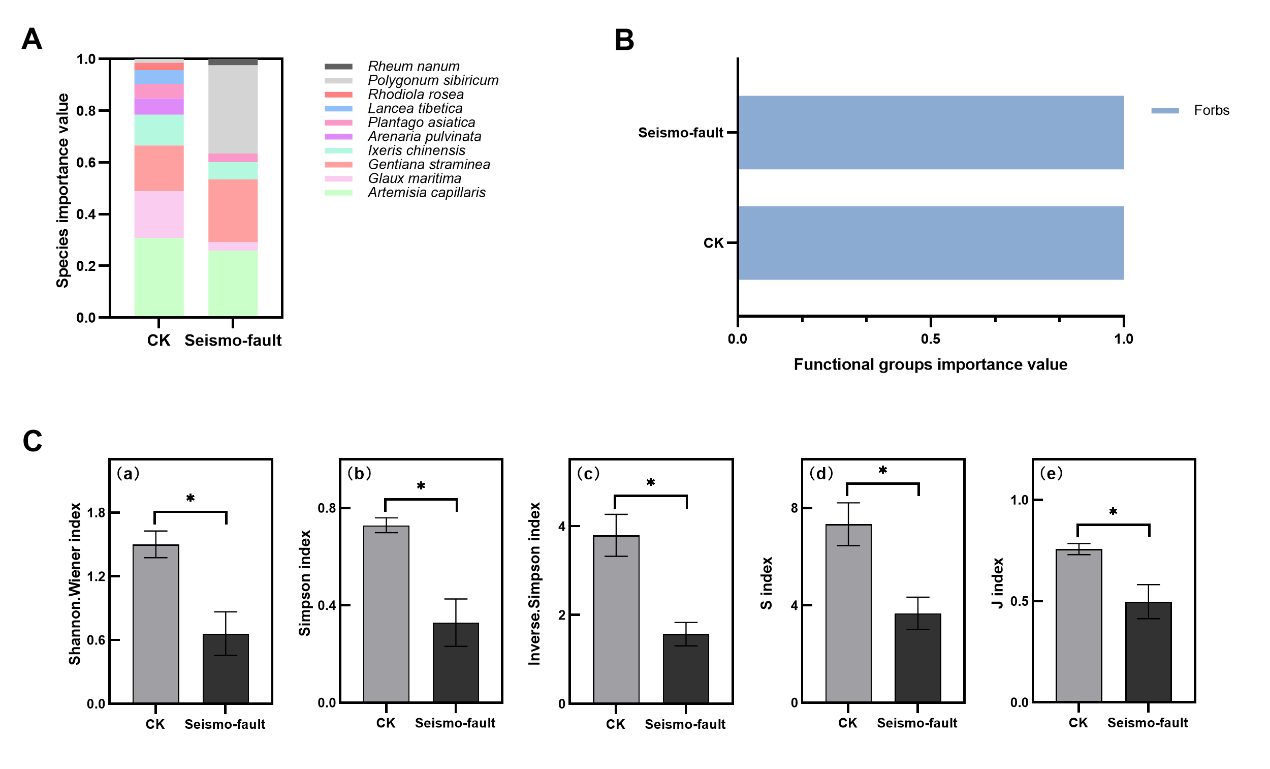


**Figure S7|** Effects of seismic faults on species and communities at sampling points Ⅵ. The **(A)** species importance values, **(B)** functional groups importance values, **(C)** diversity indices in the sampling points Ⅵ. Vertical bars represent the standard error (SE) of mean (n=3). Asterisks on the SE bars show significant differences between the control (grey bars) and seismo-fault (black bars) at **P*<0.05.


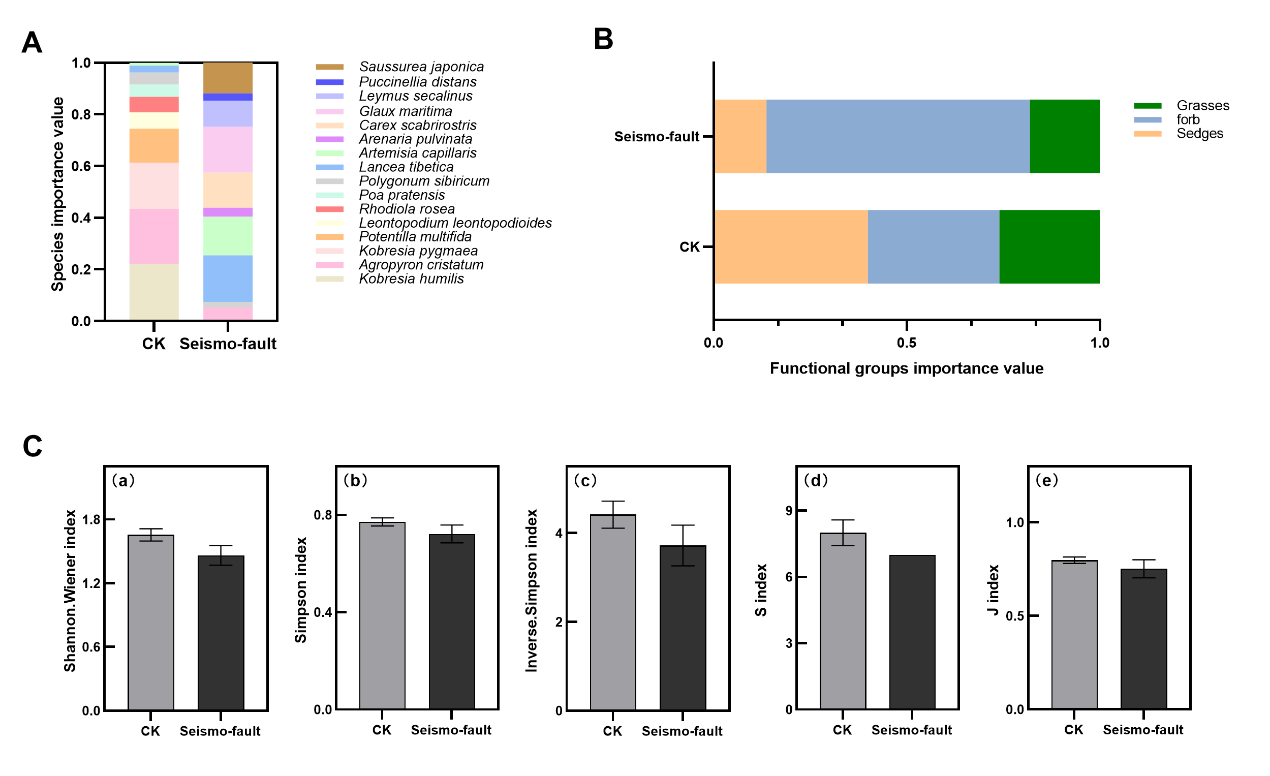


**FIGURE S8|** Effects of seismic faults on species and communities at sampling points Ⅶ. The **(A)** species importance values, **(B)** functional groups importance values, **(C)** diversity indices in the sampling points Ⅶ. Vertical bars represent the standard error (SE) of mean (n=3).

## Supplementary Tables

**Table S1|** Species composition.

| **CK** | | | |  |  | **Seismo-fault** | |  | |  |
| --- | --- | --- | --- | --- | --- | --- | --- | --- | --- | --- |
|  | **Family** | **Species** | **Life form** | **Functional group** |  | **Family** | **Species** | | **Life form** | **Functional group** |
| 1 | Gramineae | *Poa pratensis* | Perennial | Grasses | 1 | Gramineae | *Poa pratensis* | | Perennial | Grasses |
| 2 | Gramineae | *Leymus secalinus* | Perennial | Grasses | 2 | Gramineae | *Leymus secalinus* | | Perennial | Grasses |
| 3 | Gramineae | *Puccinellia distans* | Perennial | Grasses | 3 | Gramineae | *Puccinellia distans* | | Perennial | Grasses |
| 4 | Gramineae | *Agropyron cristatum* | Perennial | Grasses | 4 | Gramineae | *Agropyron cristatum* | | Perennial | Grasses |
| 5 | Cyperaceae | *Kobresia pygmaea* | Perennial | Sedges | 5 | Cyperaceae | *Kobresia pygmaea* | | Perennial | Sedges |
| 6 | Cyperaceae | *Kobresia humilis* | Perennial | Sedges | 6 | Cyperaceae | *Carex scabrirostris* | | Perennial | Sedges |
| 7 | Cyperaceae | *Kobresia tibetica* | Perennial | Sedges | 7 | Compositae | *Saussurea japonica* | | Biennial | Forbs |
| 8 | Cyperaceae | *Carex scabrirostris* | Perennial | Sedges | 8 | Compositae | *Artemisia capillaris* | | Perennial | Forbs |
| 9 | Cyperaceae | *Scirpus triqueter* | Perennial | Sedges | 9 | Compositae | *Artemisia frigida* | | Perennial | Forbs |
| 10 | Cyperaceae | *Scirpus distigmaticus* | Perennial | Sedges | 10 | Compositae | *Leontopodium leontopodioides* | | Perennial | Forbs |
| 11 | Leguminosae | *Oxytropis ochrocephala* | Perennial | Legumes | 11 | Compositae | *Cirsium japonicum* | | Perennial | Forbs |
| 12 | Compositae | *Saussurea stella* | Therophyte or Biennial | Forbs | 12 | Compositae | *Ixeris chinensis* | | Perennial | Forbs |
| 13 | Compositae | *Taraxacum mongolicum* | Perennial | Forbs | 13 | Compositae | *Aster tataricus* | | Perennial | Forbs |
| 14 | Compositae | *Heteropappus altaicus* | Perennial | Forbs | 14 | Liliaceae | *Allium przewalskianum* | | Perennial | Forbs |
| 15 | Compositae | *Artemisia capillaris* | Perennial | Forbs | 15 | Ranunculaceae | *Ranunculus membranaceus* | | Perennial | Forbs |
| 16 | Compositae | *Artemisia frigida* | Perennial | Forbs | 16 | Ranunculaceae | *Thalictrum petaloideum* | | Perennial | Forbs |
| 17 | Compositae | *Leontopodium leontopodioides* | Perennial | Forbs | 17 | Plumbaginaceae | *Limonium aureum* | | Perennial | Forbs |
| 18 | Compositae | *Cirsium japonicum* | Perennial | Forbs | 18 | Polygonaceae | *Rheum nanum* | | Perennial | Forbs |
| 19 | Compositae | *Ixeris chinensis* | Perennial | Forbs | 19 | Polygonaceae | *Polygonum sibiricum* | | Perennial | Forbs |
| 20 | Polygonaceae | *Polygonum sibiricum* | Perennial | Forbs | 20 | Gentianaceae | *Comastoma pulmonarium* | | Therophyte | Forbs |
| 21 | Gentianaceae | *Gentiana straminea* | Perennial | Forbs | 21 | Gentianaceae | *Gentiana straminea* | | Perennial | Forbs |
| 22 | Liliaceae | *Allium przewalskianum* | Perennial | Forbs | 22 | Primulaceae | *Glaux maritima* | | Perennial | Forbs |
| 23 | Liliaceae | *Allium sikkimense* | Perennial | Forbs | 23 | Caryophyllaceae | *Arenaria pulvinata* | | Perennial | Forbs |
| 24 | Primulaceae | *Glaux maritima* | Perennial | Forbs | 24 | Scrophulariaceae | *Lancea tibetica* | | Perennial | Forbs |
| 25 | Caryophyllaceae | *Arenaria pulvinata* | Perennial | Forbs | 25 | Plantaginaceae | *Plantago asiatica* | | Biennial or Perennial | Forbs |
| 26 | Scrophulariaceae | *Lancea tibetica* | Perennial | Forbs | 26 | Crassulaceae | *Rhodiola rosea* | | Perennial | Forbs |
| 27 | Scrophulariaceae | *Lagotis brachystachya* | Perennial | Forbs | 27 | Rosaceae | *Potentilla bifurca* | | Perennial | Forbs |
| 28 | Plantaginaceae | *Plantago asiatica* | Biennial or Perennial | Forbs |  |  |  | |  |  |
| 29 | Crassulaceae | *Rhodiola rosea* | Perennial | Forbs |  |  |  | |  |  |
| 30 | Rosaceae | *Potentilla bifurca* | Perennial | Forbs |  |  |  | |  |  |
| 31 | Rosaceae | *Potentilla acaulis* | Perennial | Forbs |  |  |  | |  |  |
| 32 | Rosaceae | *Potentilla multifida* | Perennial | Forbs |  |  |  | |  |  |
| 33 | Labiatae | *Dracocephalum heterophyllum* | Perennial | Forbs |  |  |  | |  |  |
| 34 | Campanulaceae | *Adenophora capillaris* | Perennial | Forbs |  |  |  | |  |  |
| 35 | Juncaginaceae | *Triglochin palustre* | Perennial | Forbs |  |  |  | |  |  |
| 36 | Juncaginaceae | *Triglochin maritimum* | Perennial | Forbs |  |  |  | |  |  |
| 37 | Araliaceae | *Oplopanax elatus* | Perennial | Others |  |  |  | |  |  |
| 38 | Suoicacae | *Salix cupularis* | perennial | Others |  |  |  | |  |  |

**TABLE S2|** Results (F values) of two-way ANOVA on the effects of earthquake fracture, different sampling sites and their interactions on community composition.

|  | **Sedges** | **Grasses** | **Legumes** | **Forbs** | **Others** |
| --- | --- | --- | --- | --- | --- |
| **Fracture** | 69.715*** | 0.162 | 6.004* | 66.111*** | 5.197* |
| **Sampling sites** | 36.658*** | 11.522*** | 2.024 | 18.558*** | 3.338* |
| **Fracture * Sampling sites** | 10.622*** | 2.474* | 2.024 | 6.356*** | 3.338* |

Note: **P*<0.05, ** *P*<0.01 and *** *P*<0.001

**TABLE S3|** Results (F values) of two-way ANOVA on the effects of earthquake fracture, different sampling sites and their interactions on diversity indices.

|  | **Shannon-Wiener** | **Simpson** | **Inverse Simpson** | **Species richness** | **Species evenness** |
| --- | --- | --- | --- | --- | --- |
| **Fracture** | 26.156*** | 16.695*** | 22.584*** | 58.018*** | 0.654 |
| **Sampling sites** | 15.892*** | 12.047*** | 29.110*** | 6.583*** | 10.372*** |
| **Fracture * Sampling sites** | 3.147* | 2.823* | 4.908** | 6.560*** | 1.171 |

Note: **P*<0.05, ** *P*<0.01 and *** *P*<0.001
